# Cardiac Regeneration and Repair in Zebrafish and Mammalian Models

**DOI:** 10.1007/s11886-025-02235-6

**Published:** 2025-06-17

**Authors:** Stanislao Igor Travisano, Ching-Ling Lien

**Affiliations:** 1 The Saban Research Institute of Children’s Hospital Los Angeles, Los Angeles, CA 90027 USA; 2 Departments of Surgery, Stem Cell Biology and Regenerative Medicine, Keck School of Medicine, University of Southern California, Los Angeles, CA 90033 USA

**Keywords:** Epicardium, Endothelial cells, Inflammation, Fibrosis, Vascularization, Cardiomyocytes

## Abstract

**Aim:**

In this review, we discuss the regenerative processes in the heart, focusing on non-cardiomyocyte cell populations (fibroblasts, immune cells, and endothelial cells) in zebrafish and mammals. We highlight the role of signaling pathways in heart repair and the potential for therapeutic strategies based on these mechanisms.

**Purpose of Review:**

The review examines key molecular and cellular mechanisms in cardiac regeneration, with a focus on fibroblasts, immune modulation, and endothelial function, to identify strategies for enhancing heart repair.

**Recent Findings:**

Recent advancements in characterization of different cell types at the single cell level, along with the discovery of regeneration enhancer elements, have opened new avenues for cardiac regeneration.

**Summary:**

Targeting the epicardium, along with fibroblast activation, immune modulation, and endothelial signaling, may offer therapeutic strategies to enhance heart regeneration by supporting cardiomyocytes in mice and humans. While non-cardiomyocytes in zebrafish contribute to heart regeneration, in mice and humans, these cells often drive fibrosis instead. Understanding these species-specific differences is crucial for optimizing therapeutic approaches to treat cardiac injury and prevent fibrosis.

**Graphical Abstract:**

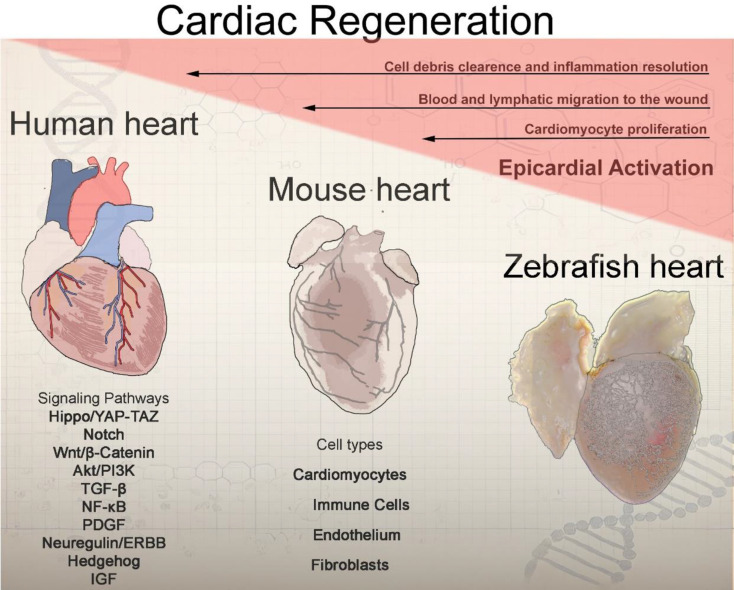

## Introduction

Cardiac regeneration is a critical area of research, with the potential to address the challenges posed by heart failure and other cardiac diseases. Zebrafish have become a key model organism due to their extraordinary regenerative capabilities, especially in the heart where different processes regulated by signaling pathways take place in the early stages of the regeneration [1]. This groundbreaking discovery demonstrating that zebrafish could fully regenerate heart tissue following amputation, ignited considerable interest in exploring heart regeneration at the cellular and molecular levels [2–4]. In addition to the apical resection model, cryoinjury was used to study the inflammatory response following myocardial infarction (MI) [5, 6]. Another method utilized the genetic ablation of cardiomyocytes to highlight their proliferative potential [7]. These studies demonstrated that the adult zebrafish heart retains substantial regenerative capacity. In mammals, heart regeneration is limited but remains an active area of research. In the neonatal mouse heart, the ability to regenerate heart tissue is transient and declines rapidly postnatally, a finding that provides a potential therapeutic window for enhancing regeneration [8]. Reprogramming non-myocytes with cardiac transcription factors has been demonstrated as a potential method to induce new cardiomyocyte formation, suggesting a promising strategy for heart repair [9, 10]. Mononuclear diploid cardiomyocytes contribute to the natural variation in regenerative capacity, highlighting their importance in myocardial replenishment [11]. Similarly, a study discovered that polyploidization of cardiomyocytes limits regeneration in zebrafish, highlighting an intrinsic constraint on regenerative capacity [12].

The regenerative process in zebrafish is supported by many non-cardiomyocyte cell populations. The epicardial cells play a key role in the response to injury and dynamic changes in the epicardium following heart injury are essential for the promotion of heart regeneration [13]. Similarly, other studies showed that *tcf21 +* epicardial cells contribute to regeneration by differentiating into non-cardiomyocyte cells and supporting tissue repair [14]. One primary contributor to cardiac regeneration is the CM population marked by a transgenic reporter driven by the *gata4* enhancer; these *gata4*+ cardiomyocytes have been extensively characterized in zebrafish [15]. Single-nucleotide variants (SNVs) within the human GATA4 enhancer increase ETS transcription factor binding affinity, providing genetic evidence for a novel transcriptional regulation of cardiomyocyte progenitor populations [16]. More recently, a Sox10 Cre labeled population contributing to myocardial regeneration has also been studied [17–19]; however the extent to which these two CM populations overlap has not yet been studied.

A critical role for cardiac innervation has also been demonstrated, as cholinergic denervation reduces myocyte proliferation, indicating that neural signaling is essential for regulating heart regeneration and myocyte repair [20]. Furthermore, the heart is composed of a diverse range of cell types beyond just cardiomyocytes (CM) [21], and exhibits a sex-specific cellular profile [22]. Based on published data, we have summarized the four main cell types (cardiomyocytes, fibroblast, endothelial and immune cells) of the heart from human, mouse and zebrafish (Table 1). Research on cardiac regeneration in both zebrafish and mammalian models emphasizes the critical roles of cardiomyocyte proliferation, epicardial progenitor cells, immune modulation, and signaling pathways. Recently, the roles of the endothelium and fibroblasts have also gained increased attention from researchers. A comparison of the ventricular cellular composition across human, mouse, and zebrafish is provided in Table 1. By integrating data from these species, researchers aim to develop more effective therapeutic strategies for repairing myocardial damage and restoring cardiac function in humans.

**Table 1:** Cell composition comparison of the human, mouse, and zebrafish hearts

Cell Type	Human (%)[21, 23]	Mouse (%)[23]	Zebrafish (%)[24]
Cardiomyocytes	~ 33–49%	~ 25–35%	~ 30–40%
Fibroblasts	~ 15–20%	~ 14%	~ 15%
Endothelial Cells	~ 10–24%	~ 40%	~ 35%
Smooth Muscle Cells	~ 5–7%	~ 5%	~ 5%
Immune Cells	~ 2–5%	~ 3–7%	~ 5–10%
Others (e.g., neuron, pericytes)	~ 2–3%	~ 5–10%	~ 5%

**Fig. 1:**
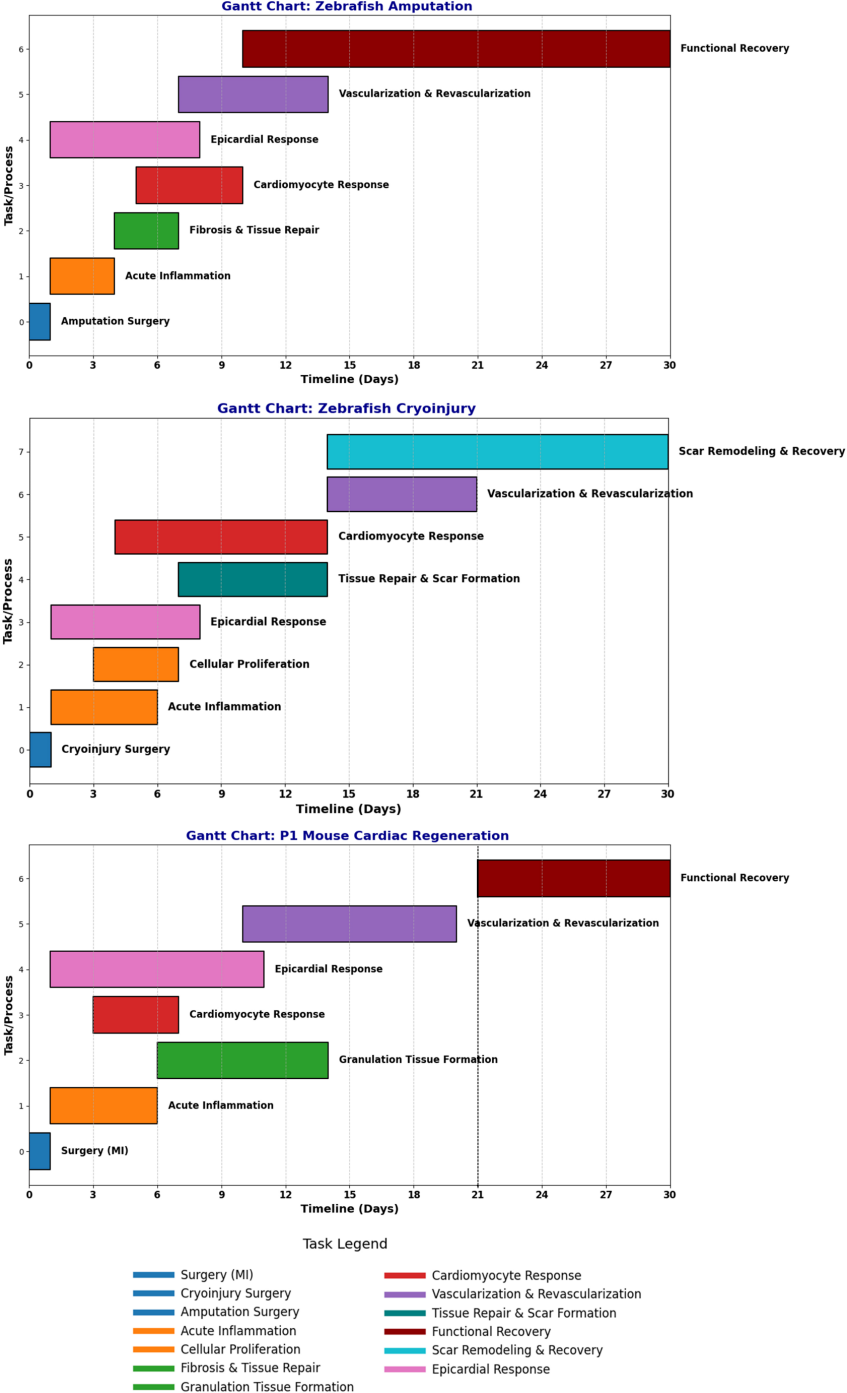
Gantt charts illustrating the processes involved in cardiac regeneration during the first 30 days in zebrafish and mice

These findings suggest that successful strategies should not only enhance CM proliferation but also address the roles of supporting cell populations to create a holistic, and functional regenerative environment. The experimental models employed—such as amputation and cryoinjury in zebrafish, and apical resection in neonatal mice—induce divergent cellular and molecular responses (Fig. 1). These differences highlight the need for a more comprehensive understanding of the cellular composition and the regulatory mechanisms involved in cardiac regeneration. Here we highlight recent advancements in characterizing these four cell types and their roles in heart regeneration.

### Epicardium and EPDCs (Epicardium Derived Cells)

The epicardium, the outermost mesothelial layer of the heart, which represent a hub for paracrine signalling during development [25]. It plays an essential role in cardiovascular repair as it is reactivated during regeneration giving rise to EPDCs that aid in tissue repair after injury and support cardiomyocytes proliferation [26–28] and revascularization [29]. In injured mouse hearts, the heterogeneity of epicardium and epicardium-derived cells, which support repair but often result in fibrosis instead of complete regeneration, has recently been revealed [30]. In zebrafish, epicardial cells are regulated by a transient program that enables complete heart regeneration [31] and promotes cardiomyocyte proliferation but is different from what is deployed during development [32]. Single-nuclei multi-omics analyses have revealed that the human fetal epicardium and subepicardium consist of distinct progenitor cells [33, 34] and the plasticity and heterogeneity of these populations may differ from the potential of adult epicardium-derived cells [35]. Complex paracrine signaling within the epicardium also involves extracellular vesicles (EVs) carrying miRNA cargo [36], suggesting that the secretion of exosome-miRNA could mediate cardiomyocyte proliferation during heart regeneration. Hapln1 in the epicardium facilitates the formation of a hyaluronic acid-containing matrix at cardiac injury sites, promoting cardiomyocyte proliferation and muscle regeneration [37]. Additionally, *ptx3a*+ epicardial cells play a crucial role in immune regulation during heart regeneration by expressing *csf1a*, which modulates the reparative macrophage response following heart injury [38]. This highlights the diverse regulatory functions of epicardial cells.

### Fibroblast

Fibroblasts play a critical role in the heart regeneration process following injury. They are involved in forming the extracellular matrix (ECM) and signaling to neighboring cells, which are essential steps for tissue repair and remodeling. Studies have shown that fibroblasts are dynamically activated during heart regeneration in zebrafish and mammals, influencing both scar tissue formation and eventual resolution of fibrosis. In zebrafish, it was demonstrated that transient fibrosis after heart injury resolves as fibroblasts undergo inactivation, allowing for the regeneration of functional cardiac tissue [39]. The ability of fibroblasts to transition between pro-fibrotic and reparative states is central to their role in cardiac repair. Similarly, Hu et al. identified distinct activated fibroblast states in zebrafish, where these cells contribute to the restoration of the myocardium while avoiding excessive fibrosis that could hinder regeneration [40]. This suggests that fibroblasts have an essential function not only in forming the scaffold for new tissue but also in regulating the extent of fibrosis to promote healing without impairing regenerative capacity. In addition to their structural role, fibroblasts also produce key signaling molecules that facilitate the proliferation and differentiation of cardiomyocytes. For example, Feng et al [41] highlighted the importance of versican, a proteoglycan produced by fibroblasts, in promoting cardiomyocyte proliferation and cardiac repair. Versican is part of the ECM, which helps to maintain tissue integrity and supports cellular migration and proliferation during the regeneration process. The interplay between fibroblasts and cardiomyocytes is therefore essential for effective heart regeneration, as fibroblasts modulate both the mechanical environment and the biochemical cues that influence cardiomyocyte behavior. Single-cell RNA sequencing of the total non-cardiomyocyte (non-CM) fractions and enriched Pdgfra-GFP + fibroblast lineage cells from murine hearts on days 3 and 7 post-injury was analyzed following MI [42]. Moreover, Hesse et al. utilized single-cell transcriptomics to define the heterogeneity of epicardial cells and fibroblasts in infarcted murine hearts, further supporting the idea that fibroblasts undergo significant functional changes post-injury [30]. These studies provide new insights into the diverse roles of fibroblasts in the injured heart, ranging from supporting tissue regeneration to contributing to the fibrotic response. Furthermore, inhibition of IL-11, a downstream effector of TGFβ1 in fibroblasts, was shown as a potential therapeutic strategy to treat cardiac fibrosis diseases [43]. However, both regenerative and fibrotic roles have been reported for Il11a in zebrafish heart regeneration, highlighting the importance of balancing these two processes [44]. 

Fibroblasts’ ability to switch between different states based on the cues they receive from the surrounding environment is key to successful heart repair. Recent studies using hPSC-based cardiomyocyte/epicardial organoids have identified fibroblast subpopulations with heterogeneity akin to that of healthy and diseased human heart populations, paving the way for targeted modulation of fibrotic responses in cardiac repair [45]. The regeneration of the heart, therefore, depends on a delicate balance between fibroblast activation and deactivation, with the cells producing both structural and biochemical signals that can either promote healing or contribute to maladaptive fibrosis. By understanding the molecular mechanisms regulating fibroblast function, particularly in the context of epicardial and myofibroblast activation, new therapeutic strategies could be developed to enhance heart regeneration in humans. During inflammatory perturbations like MI, cardiac fibroblasts rapidly switch to an inflammatory state and start to interact with infiltrating immune cells [46]. This led the scientific community to recently link cardiac fibrosis with immune modulation [47] and to emphasize the importance of focusing on the roles of cardiac fibroblasts, immune cells, the conduction system, and nervous system cell populations, rather than solely targeting the generation of new muscle and blood vessels [48]. These findings highlight the importance of fibroblasts not only as structural components of the ECM but also as active participants in the regenerative process, helping bridge the gap between myocardial injury and full recovery.

Myocardial fibrosis is triggered after MI and serves to repair the damaged heart tissue by forming fibrotic scar tissue, which helps prevent ventricular rupture. Cardiac fibroblasts are the main drivers of this fibrotic response. Upon activation, these fibroblasts secrete a variety of fibrotic factors and extracellular matrix (ECM) components, including fibronectin (FN) and collagen. These molecules accumulate in the interstitial and perivascular spaces, contributing to the formation of the scar tissue [49]. Myofibroblasts arise from the differentiation of Tcf21+ resident cardiac fibroblasts. Lineage tracing experiments of the injured murine heart identified Tcf21^+^ cells derived Periostin^+^ myofibroblasts as the cardiac fibroblasts responsible for mediating healing. These cells play a crucial role in the heart’s repair processes, particularly in scar tissue formation [50]. Macrophages are capable of secreting profibrotic cytokines such as transforming growth factor beta (TGF-β), angiotensin II, and platelet-derived growth factor (PDGF). These cytokines could trigger the activation of cardiac fibroblasts and initiate fibrotic processes [49]. Strategies aimed at inhibiting myofibroblast formation explored the use of human recombinant hepatocyte growth factor (HGF), a potent agonist of the tyrosine kinase receptor c-MET, which has been shown to effectively reduce fibrosis [51, 52].

### Immune Cells

Immune cells, particularly macrophages and T-regulatory cells (Tregs), play a pivotal role in heart regeneration by orchestrating inflammation, tissue remodeling, and cellular repair processes following cardiac injury. In zebrafish heart and spinal cord regeneration, Tregs marked by *foxp3a* expression play crucial roles in maintaining pro-regenerative capacity [53]. These findings suggest that Treg cells could be harnessed to enhance regenerative therapies, providing new insights into their potential therapeutic applications in tissue repair and regeneration [53, 54]. In mouse, Treg-cell activation not only promotes M2-like macrophage differentiation within the healing myocardium, but also triggers myofibroblast activation and enhances the expression of monocyte/macrophage-derived proteins that support wound healing [55]. In both zebrafish and mammals, macrophages are involved in the clearance of damaged tissue and the promotion of tissue repair. In zebrafish, macrophages not only clear dead cells but also contribute directly to collagen deposition in the regenerating heart, thereby assisting in the formation of a scaffold for new tissue [56]. This collagen contribution is crucial for maintaining the structural integrity of the regenerating myocardium, underscoring the macrophages’ multifaceted role in repair. Furthermore, a recent comparative study showed heterogeneity of macrophages in zebrafish and essential roles of resident macrophages for efficient heart regeneration that cannot be replaced by circulating macrophages [57].

Macrophages also exhibit a dynamic response to injury, transitioning between pro-inflammatory and pro-healing states depending on the stage of regeneration. In neonatal mammals, macrophages are required for heart regeneration, with these cells promoting a pro-regenerative environment by releasing cytokines and growth factors that stimulate cardiomyocyte proliferation [58]. In contrast in adult hearts, macrophages’ roles are more complex, as they contribute to both healing and fibrosis, depending on their polarization. The interplay between macrophage activation and the resolution of inflammation is crucial for preventing excessive scar tissue formation, which can hinder regenerative processes [59]. They further elucidated the role of distinct macrophage lineages in the neonatal and adult heart, revealing that resident macrophages in the neonatal heart promote efficient repair and regeneration, while in the adult heart, the monocytes derived macrophages tend to favour scar formation and remodeling, leading to more limited regeneration.

Antigen presentation also plays a positive role in cardiac regeneration, particularly in zebrafish [60]. This process involves activation of endocardial cells and other immune cells and Cd74 expression, supporting a coordinated response to cardiac injury. In the meantime, the role of interleukin-11 (IL-11) in modulating macrophage to myofibroblast transition and cardiac fibrosis was also explored [61]. These studies suggest that targeting immune modulation could offer a potential therapeutic strategy to boost heart regeneration in mammals. These studies also highlight the crucial role of macrophages in heart regeneration, not only for clearing debris but also for actively participating in tissue repair and remodeling. Maintaining a balance between macrophage-driven inflammation and healing is key to promoting regeneration while preventing maladaptive fibrosis, offering valuable insights into potential therapies that harness the immune system’s regenerative capabilities to repair damaged hearts. Beside the macrophages role, following MI, a marked infiltration of neutrophils occurs in mice, and it has traditionally been postulated that these cells contribute to worsening cardiac injury [62]. However, a recent investigation provided compelling evidence that depleting neutrophils impairs heart function, accelerates fibrosis, and promotes the onset of heart failure. This study further revealed that neutrophil depletion disrupted the balance of macrophage polarization, suggesting that neutrophils play a crucial, previously underestimated role in regulating the inflammatory and repair processes following MI [63].

These findings suggest that cardiomyocytes and resident macrophages play a role in ECM remodeling at the border zone, facilitating cardiomyocyte replenishment in the fibrotic injured tissue. This process, which appears crucial for scar resolution in the zebrafish heart, highlights the importance of collagenolytic activity [64]. However, this mechanism is insufficient in mouse hearts, which lack efficient myocardial regeneration.

### Blood and Lymphatic Endothelium

The vascular system, consisting of both blood and lymphatic endothelium, plays a central role in health and disease [65, 66]. The role of the endothelium facilitating the rapid formation of new blood vessels and restoring tissue homeostasis following injury has extensively been reviewed [67]. Regulation of the tissue microenvironment byblood and lymphatic vascuature across different organs is essential for normal tissue development, homeostasis, and regeneration after injury [68]. Its heterogeneity plays a key role in the pathophysiology of conditions such as inflammation and cancer [69]. The dynamic regulation of angiogenesis and lymphangiogenesis during heart regeneration is crucial for the timely repair of the damaged myocardium. 

Coronary vasculature forms in zebrafish through angiogenesis regulated by Cxcl12 chemokine signaling and fish with a mutation in Cxcr4a receptor fail to regenerate their hearts [70]. During neonatal mouse heart regeneration, endothelial cells migrate towards the apex after resection and form arteries, and this precedes cardiomyocyte migration into the the regenerating area, highlighting the importance of rapid angiogenesis [71]. The process of coronary revascularization is conserved and tightly coupled with myocardial regeneration, and endothelial cells contribute significantly to tissue repair with new blood vessels providing critical support for cardiomyocyte repopulation. The formation of a functional vascular network in the injured heart not only provides necessary nutrients and oxygen but also acts as a scaffold for cardiomyocyte regeneration; and inducing collateral arteries enhances heart regeneration [72–74]. Hapln1a + cells and serpine1 play a central role in creating a microenvironment that guides coronary growth, with specific signaling cues required to regulate hyaluronan during zebrafish heart regeneration [75].

In addition to blood vessels, the lymphatic system contributes to cardiac repair by facilitating the clearance of cellular debris and signaling to other regenerative cells. Mouse cardiac lymphatic endothelium responds to injury and promote cardiac functional recovery [76]. The cardiac lymphatic vasculature in zebrafish plays a pivotal role in maintaining heart function and facilitating regeneration by modulating interstitial fluid balance and promoting immune cell clearance [77, 78]. Signals from mouse lymphatic endothelial cells, termed “lymphoangiocrines” (such as Reelin) are important secreted proteins which promote both cardiac growth and repair. These signals modulate the behavior of nearby cells, including cardiomyocytes, thereby enhancing tissue regeneration [79]. The interplay with blood and lymphatic vessels is critical for successful heart regeneration, as it ensures efficient delivery of oxygen and nutrients and a coordinated response to injury that involves both vascular and immune components. A population of cardiac lymphatic endothelial cells expressing PROX1 and RELN and associated with the coronary artery was recently identified in the human fetal heart [34]. This association is similar to what is observed in zebrafish but distinct from mice where lymphatics are mainly associated with coronary veins [80]. Furthermore, the presence of cardiac lymphatics in both health and disease underscores the critical role of these vessels not only in maintaining tissue homeostasis but also in promoting effective repair after injury [81]. Targeting both angiogenesis and lymphangiogenesis may offer new therapeutic avenues to enhance heart regeneration in mammals, where revascularization is often a bottleneck in healing after MI.

In sum, the vascular system, encompassing both blood and lymphatic endothelium, is integral to heart regeneration. The development of a robust vascular network is essential for delivering the nutrients required for tissue growth, resolving inflammation, and supporting the regeneration of cardiomyocytes. The signaling of Interleukin-11 on endothelial cells promotes cellular reprogramming and reduces fibrotic scarring during tissue regeneration [82]. It is now clear that a deeper understanding of the fundamental differences in signaling pathways between regenerative and non-regenerative species, as well as across various cell types, will be essential for developing effective regenerative and antifibrotic therapies. As demonstrated with TGF-β, the pleiotropic role of IL-11 highlights the importance of personalized therapeutic strategies following MI. A thorough understanding of the timing of each process post-MI, along with precise targeting of specific cell types, is crucial for optimizing cardiac repair and minimizing potential side effects.

### Endocardium

The endocardium lines the heart chambers and shares molecular markers with the coronary endothelium, making it difficult to differentiate their contributions during heart regeneration. The endocardium has been identified as another source of fibroblast and fibrosis in different cardiac injury setting [40, 83]. Moreover, it provides another critical signaling cue that influences inflammation and fibrosis during tissue regeneration, while also playing a role in regulating cardiomyocyte proliferation [84, 85].

### Heart Regeneration Enhancer Element Drives Candidate Gene Expression

While significant progress has been made in understanding the role of signaling pathways in cardiac regeneration, comprehensive characterization of the chromatin landscape across different cell types remains largely unexplored. Due to the systemic effects of NRG1 administration therapy, an enhancer-based strategy to locally activate gene expression via the tissue-regeneration enhancer elements (TREEs) could potentially ensure improved cardiac regeneration [86]. Recent epigenetic profiling has identified a short DNA sequence upstream of *lepb*, called *lepb*-linked enhancer (*LEN*) that drives gene expression in the injury sites of the regenerating zebrafish hearts [86]. Profiling the replacement of H3.3 histone, which are typically deposited in regions of the genome undergoing active nucleosome turnover provids key insights into the gene regulatory changes that occurs during cardiac regeneration [87]. Another DNA element, the careg element, which regulates regeneration in the zebrafish myocardium and depends on TGFβ/ActivinB signaling, was characterized [88]. Three different TREEs identified in zebrafish cardiac regeneration, the LEN, the runx1-linked enhancer (REN), and the il11a-linked enhancer (il11aEN), were delivered via recombinant AAV vectors into injured hearts of mice and two into pigs. These data also suggest that TREEs can be recognized by evolutionarily conserved transcriptional machinery to drive temporospatial specific gene expression. This study demonstrates a potential application of using TREE-based gene therapy vectors to control gene expression in regenerative medicine [89].

## Future Directions and Conclusion

The field of cardiac regeneration has made significant strides, particularly through the study of zebrafish and their remarkable ability to regenerate heart tissue. However, translating these findings to mammalian models, especially humans, remains a formidable challenge. Future research should focus on several key areas to bridge this gap and develop effective therapeutic strategies for heart regeneration.

In conclusion, the future of cardiac regeneration research lies in integrating findings from various model organisms and cell types to develop comprehensive, multi-targeted therapies. By enhancing cardiomyocyte proliferation, modulating the immune response, regulating fibroblast activity, and supporting vascular and lymphatic systems, we can move closer to achieving effective heart regeneration in humans. The ultimate goal is to develop personalized therapeutic strategies that optimize cardiac repair and minimize potential side effects, paving the way for improved outcomes in patients with heart failure and other cardiac diseases.

## Key References

- Weinberger M, Simões FC, Gungoosingh T, Sauka-Spengler T, Riley PR. Distinct epicardial gene regulatory programs drive development and regeneration of the zebrafish heart. Dev Cell [Internet]. 2024;59:351–367.e6. Available from: https://www.sciencedirect.com/science/article/pii/S1534580723006925.
-
  - This study identifies distinct genetic programs driving epicardial development and regeneration in zebrafish, revealing that heart regeneration involves more than just reactivating developmental pathways.
- Travisano SI, Harrison MRM, Thornton ME, Grubbs BH, Quertermous T, Lien C-L. Single-nuclei multiomic analyses identify human cardiac lymphatic endothelial cells associated with coronary arteries in the epicardium. Cell Rep [Internet]. 2023;42. Available from: 10.1016/j.celrep.2023.113106.
-
  - This work provides a single nuclei Multiomic characterization of the cells residing in the human fetal epicardium.
- Yan R, Cigliola V, Oonk KA, Petrover Z, DeLuca S, Wolfson DW, et al. An enhancer-based gene-therapy strategy for spatiotemporal control of cargoes during tissue repair. Cell Stem Cell. 2023;30:96–111.e6.
-
  - This work shows that enhancer elements discovered from zebrafish control of gene expression, enhancing cardiac regeneration in mammals.

## Data Availability

No datasets were generated or analysed during the current study.
